# 5-O-Demethylnobiletin Alleviates CCl_4_-Induced Acute Liver Injury by Equilibrating ROS-Mediated Apoptosis and Autophagy Induction

**DOI:** 10.3390/ijms22031083

**Published:** 2021-01-22

**Authors:** Sukkum Ngullie Chang, Se Ho Kim, Debasish Kumar Dey, Seon Min Park, Omaima Nasif, Vivek K. Bajpai, Sun Chul Kang, Jintae Lee, Jae Gyu Park

**Affiliations:** 1 Department of Biotechnology, Daegu University, Gyeongsan 38453, Korea; sukkumchang@gmail.com (S.N.C.); deepdey1993@daegu.ac.kr (D.K.D.); sckang@daegu.ac.kr (S.C.K.); 2 Advanced Bio Convergence Center (ABCC), Pohang Technopark Foundation, Pohang 37668, Korea; rlatpgh007@naver.com (S.H.K.); seonmin@ptp.or.kr (S.M.P.); 3 School of Chemical Engineering, Yeungnam University, Gyeongsan 38541, Korea; 4 Department of Physiology, College of Medicine, King Saud University (Medical City), King Khalid University Hospital, P.O. Box 2925, Riyadh 11461, Saudi Arabia; onasif@ksu.edu.sa; 5 Department of Energy and Materials Engineering, Dongguk University-Seoul, 30 Pildong-ro 1-gil, Seoul 04620, Korea

**Keywords:** 5-O-demethylnobiletin (5-DN), reactive oxygen species (ROS), inflammation, fibrosis, cytochrome P450, MAP kinase, apoptosis, autophagy

## Abstract

Polymethoxyflavanoids (PMFs) have exhibited a vast array of therapeutic biological properties. 5-O-Demethylnobiletin (5-DN) is one such PMF having anti-inflammatory activity, yet its role in hepatoprotection has not been studied before. Results from in vitro study revealed that 5-DN did not exert a high level of cytotoxicity on HepG2 cells at 40 μM, and it was able to rescue HepG2 cell death induced by carbon tetrachloride (CCl_4_). Subsequently, we investigated acute liver injury on BALB/c mice induced by CCl_4_ through the intraperitoneal injection of 1 mL/kg CCl_4_ and co-administration of 5-DN at (1 and 2 mg/kg) by oral gavage for 15 days. The results illustrated that treatment with 5-DN attenuated CCl_4_-induced elevated serum aminotransferase (AST)/alanine aminotransferase (ALT) ratio and significantly ameliorated severe hepatic damage such as inflammation and fibrosis evidenced through lesser aberrations in the liver histology of 5-DN dose groups. Additionally, 5-DN efficiently counteracted and equilibrated the production of ROS accelerated by CCl_4_ and dramatically downregulated the expression of CYP2E1 vitally involved in converting CCl_4_ to toxic free radicals and also enhanced the antioxidant enzymes. 5-DN treatment also inhibited cell proliferation and inflammatory pathway abnormally regulated by CCl_4_ treatment. Furthermore, the apoptotic response induced by CCl_4_ treatment was remarkably reduced by enhanced Bcl-2 expression and noticeable reduction in Bax, Bid, cleaved caspase 3, caspase 9, and apaf-1 expression. 5-DN treatment also induced the conversion of LC3 and promoted the autophagic flux. Conclusively, 5-DN exhibited hepatoprotective effects in vitro and in vivo and prevented liver fibrosis induced by CCl_4_.

## 1. Introduction

Liver is the major organ where chemicals and drugs are processed and metabolized [1]. Damage to the liver poses a serious and fatal risk. Liver fibrosis has been one of the critical issues in the medical community because of the significant cases of morbidity and mortality worldwide. Medical negligence associated with these alterations leads to fibrosis, progressing to cirrhosis, carcinoma, liver failure, and death. The major causes of liver fibrosis include chronic ethanol consumption, non-alcoholic steatohepatitis, viral infections such as hepatitis B and hepatitis C, helminthes infection, excessive amount of iron and copper in the body, obstruction to the bile ducts, and drug-induced liver damage [2,3]. A fibrotic liver is distinguished by the excessive deposition of extracellular matrix (ECM) proteins namely glycoproteins, proteoglycans, and collagens [4,5]. The most commonly studied marker for fibrosis is the accumulation of collagens, and specifically the fibrillar type of collagens. As a result of the alteration in the collagen synthesis extending from the mRNA to the protein levels, there is a hyper elevated increase in the different types of collagen and a significant recognizable increase from basal level in type III collagen [6,7]. During stimulus to fibrogen or liver injury, the hepatic stellate cells (HSCs) go through transformation in the complex and activation, which contributes to excessive collagen synthesis and other aberrations normally seen in liver fibrosis. Studies have shown that HSC transitions from a quiescent vitamin A storing cell into an activated myofibroblast-like cell [8]. The molecular changes occurring in the cells include morphological alterations such as the appearance of cytoskeleton protein smooth muscle α-actin (α-SMA) and a drastic decrease of vitamin A and increased expression of the rough endoplasmic reticulum [9,10].

We used carbon tetrachloride (CCl_4_), which is a well-known established chemical hepatotoxin to induce liver injury and fibrosis on BALB/c mice, and it has been extensively studied over the years [11]. CCl_4_ causes injury to the liver through different mechanisms such as oxidative stress, inflammation, and programmed cell death [12,13]. CCl_4_ is converted to tri-chloromethyl (CCl_3_) free radical or tri-chloroperoxyl radical (CCl_3_O_2_) produced by the cytochrome P450 oxygenase system arising from the endoplasmic reticulum [14]. The newly formed CCl_3_ free radical interacts with nucleic acids, lipids, and proteins, damaging the important cellular processes and consequently resulting in impaired lipogenesis leading to fatty acid degeneration and steatosis as well as transcribing low quantities of proteins. Furthermore, compounds formed by the addition of CCl_3_ trigger mutations in DNA synthesis, and eventually, it leads to hepatocellular carcinoma. The formed CCl_3_O_2_ free radical through the oxygenation of CCl_3_ triggers lipid peroxidation, resulting in the damage of polyunsaturated fatty acids (PUFAs). Consequently, the cell membrane becomes permeable, exposing the organelles susceptible to damage, thereby causing hepatic damage through inflammatory response, fibrosis, cirrhosis, and progression to hepatocellular carcinoma [15].

Flavonoids are a class of polyphenolic metabolites possessing a benzo-γ-pyrone structure and are abundantly found in plants. The word flavonoid comes from the Latin word flavus, which describes a natural compound that is yellow-colored by nature. There is an approximate estimation that there are around 9000 flavonoids found in different kinds of plants [16]. Flavonoids have been shown to possess several health-promoting effects because of their high antioxidant capacity, anti-bacterial, anti-inflammatory, anti-viral, anti-cancer, and hepatoprotective properties [17]. Different flavonoids extracted from citrus fruits have shown potent hepatoprotective effects on different liver disease models [18,19,20]. 5-DN is one such flavonoid commonly extracted from the citrus fruits and has been shown to possess anti-inflammatory and anticancer properties [21]. However, the hepatoprotective effect of flavonoid, 5-DN has not been studied before. Therefore, the present study was designed to evaluate the effect of flavonoid, 5-O-demethylnobiletin (5-DN) on CCl_4_-induced hepatotoxicity. We observed that 5-DN significantly reduced the accumulation of collagen, and neutralized oxidative stress arising from the CCl_4_ intermediates observed through ROS downregulation and by boosting antioxidant response. 5-DN was also able to prevent CCl_4_-induced apoptosis and subsequently activated autophagy. Taken together, 5-DN has the potential to ameliorate liver fibrosis.

## 2. Results and Discussion

### 2.1. 5-O-Demethylnobiletin Protects against CCl4-Induced Cell Death in HepG2 Cells

**Figure 1:**
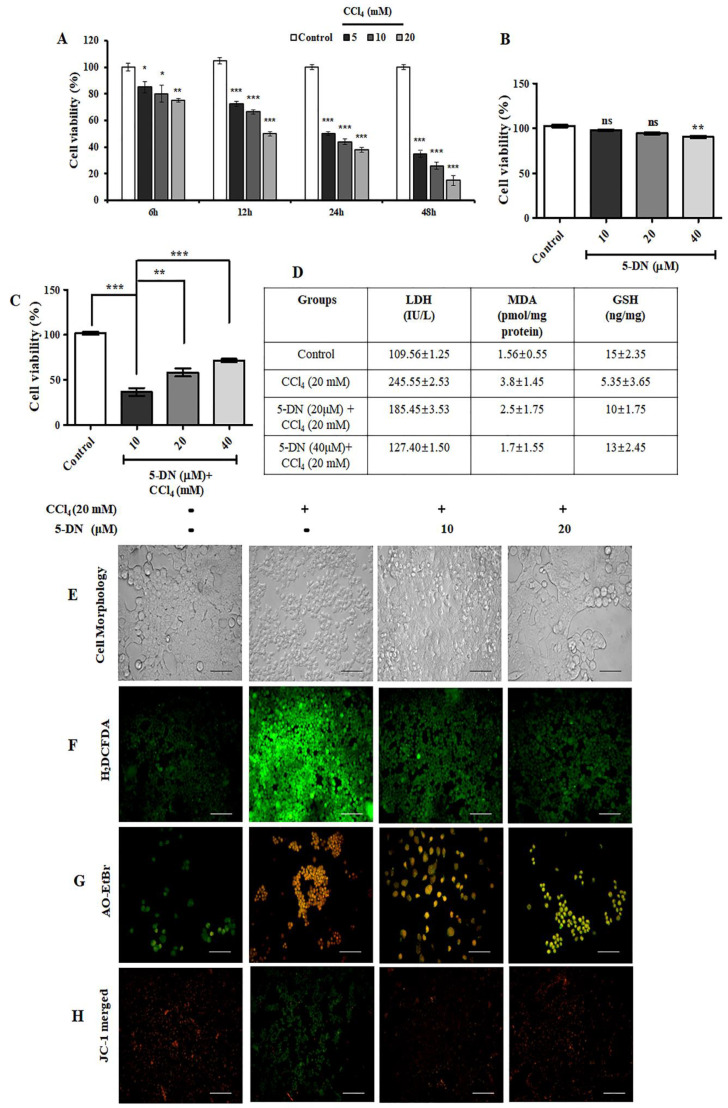
5-O-Demethylnobiletin (5-DN) rescued HepG2 cells from carbon tetrachloride (CCl_4_)-induced cell death. (**A**) 3-(4,5-Dimethylthiazol-2-yl)-2,5-diphenyltetrazolium bromide (MTT) assay at different time points after varying doses of CCl_4_ (5, 10, 20 mM). (**B**) MTT assay after 24 h treatment to HepG2 cells with different doses of 5-DN (10, 20, 40 μM). (**C**) MTT assay after co-treatment with CCl_4_ (20 mM) and 5-DN (10, 20, 40 μM). (**D**) Estimation of LDH, malondialdehyde (MDA), and GSH from HepG2 cell homogenates. (**E**) Cell morphology of HepG2 cells after 24 h co-treatment with CCl_4_ and 5-DN. (**F**) H_2_DCFDA staining of HepG2 cells. (**G**) AO-EtBr staining of HepG2 cells after 24 h co-treatment with CCl_4_ and 5-DN. (**H**) JC-1 staining of HepG2 cells after 24 h co-treatment with CCl_4_ and 5-DN. Magnification: 200×, Scale bar (100 μM). Images were captured using Olympus BX51 fluorescence microscope. The data represented here are the mean ± S.D. from three independent experiments where * *p* < 0.05, ** *p <* 0.01, *** *p* < 0.001, Control vs CCl4, CCl_4_ vs. 5–DN (1–2 mg/kg). Statistical significance analysis was carried out through one-way analysis of variance (ANOVA) prism.

We evaluated the toxicity of CCl_4_ on HepG2 cells in a time-dependent and dose-dependent manner. The results illustrated the toxic nature of CCl_4_ at a dose range (5–20 mM), and it was found to be highly cytotoxic. Cell viability massively plummeted at all-time points of 6, 12, 24, and 48 h (Figure 1A). In contrast, we observed that 5-DN was not toxic to HepG2 cells at (10, 20, 40 μM), showing a cell viability of 98%, 94.33%, and 90.66%, respectively, as shown in Figure 1B. Additionally, we co-treated HepG2 cells with CCl_4_ and 5-DN and observed that 5-DN exerted a cytoprotective effect with increased dose (Figure 1C). Likewise, the CCl_4_-treated group had an elevated expression of lactate dehydrogenase (LDH), which was significantly downregulated on CCl_4_ and 5-DN co-treatment groups (Figure 1D). Evaluation of the morphological image revealed that CCl_4_ drastically distorted the cellular morphology and caused cell death, which was observably prevented upon 5-DN treatment (Figure 1E). HepG2 cells treated with CCl_4_ also had a higher expression of malondialdehyde (MDA) (Figure 1D), ROS generation, mitochondrial membrane damage, and a higher occurrence of apoptotic event, and these effects were efficaciously prevented on cells co-treated with 5-DN (Figure 1F–H). The expression of glutathione (GSH) was also found to be relatively higher in CCl_4_ and 5-DN co-treatment groups in comparison to CCl_4_-only treatment groups (Figure 1D). In summary, 5-DN rescued HepG2 cells from CCl_4_ induced cell death and increased the expression of GSH, which is the chief antioxidant involved in the detoxification of xenobiotics and endogenous compounds.

### 2.2. 5-O-Demethylnobiletin Ameliorates CCl_4_-Induced Collagen Deposition and Severe Hepatic Injury in Mice

**Figure 2:**
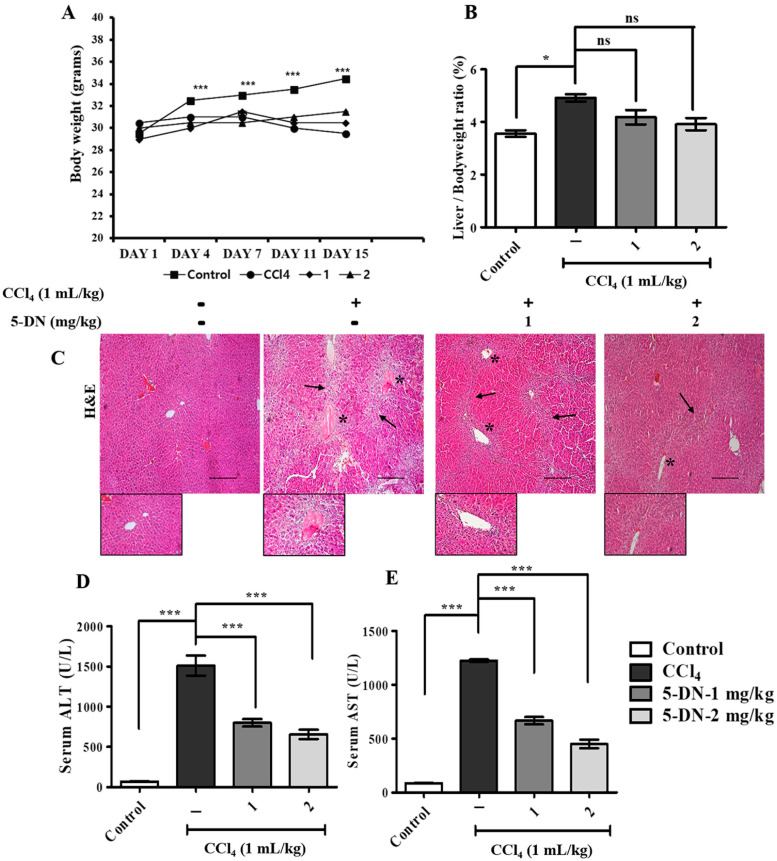
5-DN attenuated CCl_4_-induced severe hepatic damage in BALB/c mice (mice, n = 6/group). (**A**) Assessment of the body weight from different mice groups over a period of 15 days. (**B**) Liver weight/ body weight ratio in percentage of different mice groups. (**C**) H&E staining on liver tissues. Asterisk (*): represents the regenerative nodules, Arrow (→): represents the fibrous septa extending from the veins, and zoomed images represent infiltration of inflammatory cells from central veins. (**D**) Serum ALT analysis. (**E**) Serum AST analysis. Magnification: 200×, Scale bar (100 μM). Images were captured using Olympus BX51 fluorescence microscope. The data are represented here are the mean ± S.D. from three independent experiments where * *p* < 0.05, *** *p* < 0.001, Control vs. CCl_4_, CCl_4_ vs. 5–DN (1–2 mg/kg). Statistical significance analysis was carried out through one-way analysis of variance (ANOVA) prism.

**Figure 3:**
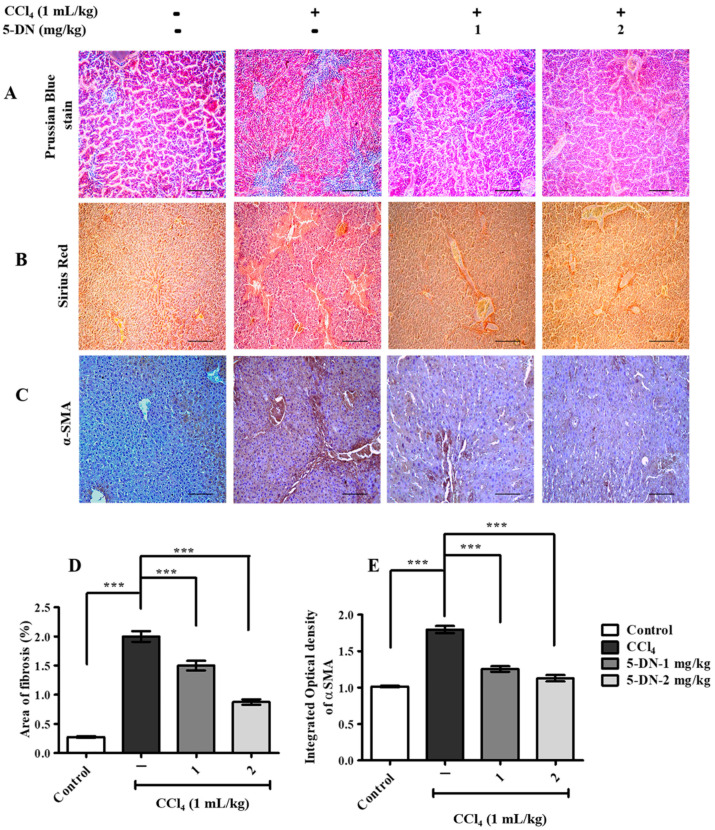
5-DN prevented BALB/c mice from CCl_4_-induced severe hepatic fibrotic damage (mice, n = 6/group). (**A**) Prussian blue staining for the detection of iron deposits in liver tissues. (**B**) Sirius Red staining for the detection of collagen. (**C**) Immunohistochemical (IHC) staining of smooth muscle α-actin (αSMA). (**D**) Percentage of fibrosis evaluated through Sirius red staining (**E**) Integrated optical density measurement of αSMA through IHC. Magnification: 200×, Scale bar (100 μM). Images were captured using Olympus BX51 fluorescence microscope. The data are represented as mean ± S.D. of three independent experiments, *** *p* < 0.001 and ns (non-significant). Control vs. CCl_4_, CCl_4_ vs. 5–DN (1–2 mg/kg). Statistical significance analysis was carried out through one-way analysis of variance (ANOVA) prism.

CCl_4_ is a well-known hepatotoxin that causes serious liver injury and cell death through death pathways such as apoptosis and necrosis [22], and prolonged exposure to CCl_4_ leads to cirrhosis and hepatocellular carcinoma [23]. We evaluated the therapeutic effect of 5-DN on CCl_4_-induced liver damage. To do this, BALB/c male mice were subjected to 3 times/week treatment with CCl_4_ alone or in co-treatment with 1–2 mg/kg of 5-DN for 15 days. We measured the body weight and calculated the liver-to-body weight ratio to observe whether the CCl_4_ group had a decreasing effect on body weight and liver weight (Figure 2A,B). Similarly, the estimation of important serum parameters such as AST and ALT revealed elevated levels in comparison to control and 5-DN treated group weight (Figure 2D,E). Histopathological analysis revealed that CCl_4_ adversely aggravated liver through the loss in the architecture, massive mononuclear cellular infiltration, inflammation, regenerative nodules, and fibrous septa (Figure 2C). In addition, CCl_4_ treatment groups had elevated levels of iron deposits (Figure 3A) evaluated in the hepatocytes as a soluble compound called ferritin or in an insoluble form known as hemosiderin. Coherently, we quantified the occurrence of collagen fiber deposits and found that CCl_4_ treatment groups had a higher expression of fibrous septa that vastly extended from the portal veins to the hepatic lobules (Figure 3B,D). In addition, immunohistochemical staining for an important marker of hepatic stellate cell activation αSMA (Figure 3C,E) revealed that CCl_4_ aggravated the liver homeostasis, which was observably attenuated by 5-DN treatment.

### 2.3. 5-O-Demethylnobiletin Ameliorates CCl4-Induced Liver Injury by Governing Inflammation and MAP Kinase Pathway

**Figure 4:**
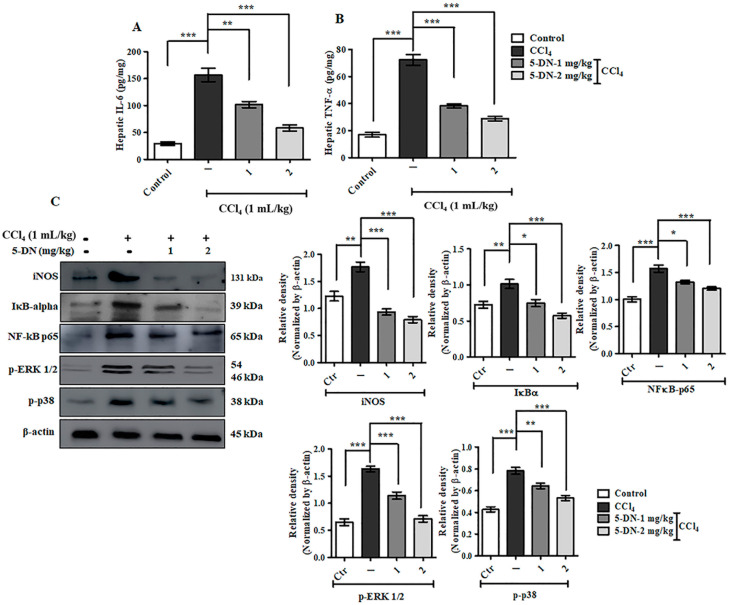
5-DN inhibited CCl_4_-induced inflammatory pathway and normalized upregulated cell proliferation pathway (mice, n = 6/group). (**A**) Estimation of hepatic IL-6. (**B**) Estimation of hepatic TNF-α. (**C**) Western blotting analysis of inflammatory and MAP kinase pathway markers. Densitometry analysis of these respective proteins were normalized by β-actin and evaluated through Image J software. The data are represented as mean ± S.D. of three independent experiments * *p* < 0.05, ** *p* < 0.01, *** *p* < 0.001 and ns (non-significant). Control vs CCl_4_, CCl_4_ vs. 5-DN (1–2 mg/kg). Statistical significance analysis was carried out through a one-way analysis of variance (ANOVA) prism.

As we know, inflammatory stimuli activate the cells of the immune system through different intracellular signaling pathways for activating and releasing the inflammatory mediators such as cytokines tumor necrosis factor- alpha (TNF-α) and interleukin- 6 (IL-6) that act through different surface receptors such as Toll-like receptors (TLRs) and activate the mitogen-activated protein kinases (MAPK) [24], thereby leading to the activation of the nuclear factor kappa- light- chain- enhancer of activated B cells (NF-κB) pathway [25]. We observed an elevated concentration of pro-inflammatory cytokines, namely TNF-α and IL-6, after treatment with CCl_4_ on the liver tissues, which was significantly neutralized after treatment with 5-DN (Figure 4A,B). In addition, IκBα and NF-κB-p65 were perceptibly reduced on 5-DN groups in comparison to CCl_4_ treatment groups (Figure 4C). Similarly, inhibiting the MAP kinase pathway has been one molecular target for ameliorating inflammatory diseases [26]. The MAP kinase family markers such as extracellular signal-regulated kinase (ERK) and p38 were found to be upregulated. When these molecules are activated, they recruit Ras, which leads to the transcription of cell proliferative markers and other pro-fibrogenic factors. MAP kinase is critically and fundamentally involved in activating the hepatic stellate cells (HSCs) and for the synthesis of collagen. Suppressing these molecular markers is associated with inhibiting the HSCs proliferation [27,28,29]. Studies have also shown that MAP kinase is associated with the progression and generation of oxidative stress [24]. Consistent with the anti-inflammatory effects of 5-DN, we observed that the MAP kinase signaling pathway was also significantly inhibited, which was evaluated through immunoblotting analysis. The expressions of p-p38 and p-ERK 1/2 were significantly downregulated (Figure 4C), which were observably and noticeably upregulated on CCl_4_ treatment groups. In conclusion, 5-DN treatment neutralized the CCl_4_-induced inflammatory response and normalized the cell proliferation pathway.

### 2.4. 5-O-Demethylnobiletin Modulates CCl_4_-Induced ROS Production by Downregulating Lipid Peroxidation, CYP2E1, and Boosting Antioxidant Enzymes

**Figure 5:**
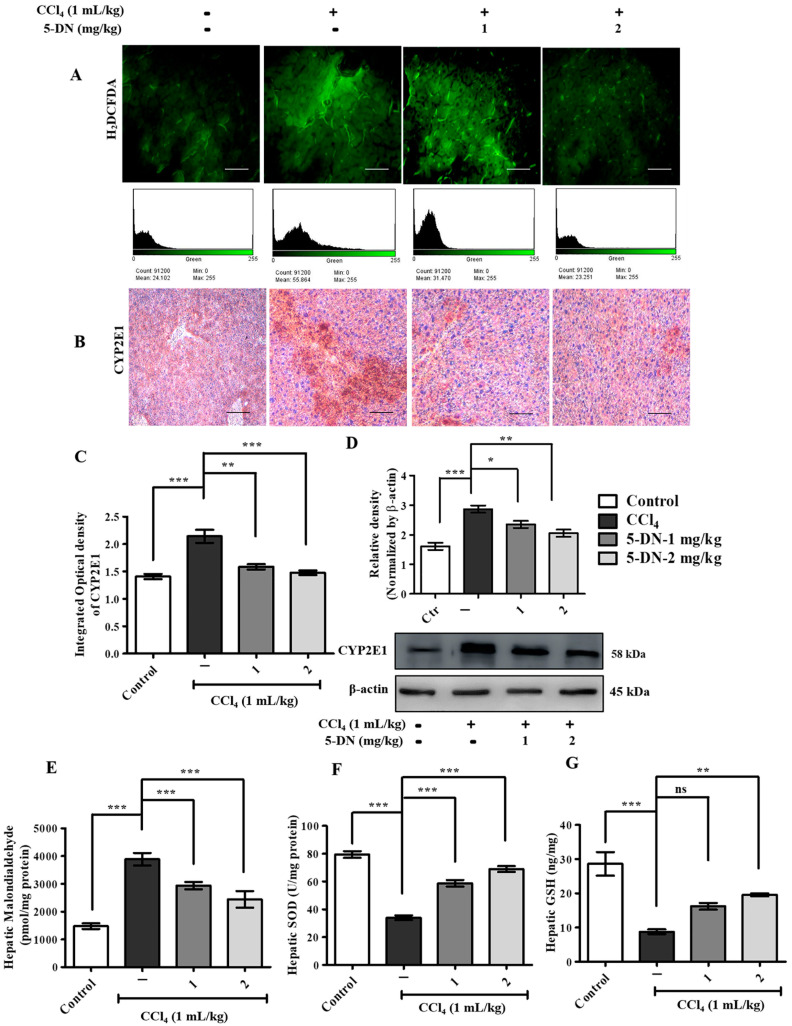
5-DN prevented CCl_4_-induced oxidative injury by inhibiting the expression of MDA, CYP2E1 boosted antioxidant enzymes (mice, n = 6/group). (**A**) H_2_DCFDA staining of liver tissues and histogram. (**B**) Immunohistochemical staining of CYP2E1. (**C**) Integrated optical density of CYP2E1. (**D**) Western blotting analysis of CYP2E1. (**E**) Hepatic malondialdehyde assay analysis. (**F**) Hepatic Superoxide dismutase assay analysis. (**G**) Hepatic glutathione assay analysis. Magnification: 200×, Scale bar (100 μM). Images were captured using Olympus BX51 fluorescence microscope. Densitometry analysis of these respective proteins were normalized by β-actin and evaluated through Image J software. The data are represented as mean ± S.D. of three independent experiments * *p* < 0.05, ** *p* < 0.01, *** *p* < 0.001 and ns (non-significant). Control vs. CCl_4_, CCl_4_ vs. 5–DN (1–2 mg/kg). Statistical significance analysis was carried out through one-way analysis of variance (ANOVA) prism.

5-DN is a citrus flavonoid possessing high antioxidant activity, which directly participates in reducing the oxidative stress induced by CCl_4_ [30]. An excessive amount of endogenous ROS generation creates an imbalance in the redox reaction, consequently increasing the oxidative stress, which resultantly damages the important biomolecules necessary for cell survival [31]. The toxic effects of CCl_4_ arise from the conversion of CCl_4_ molecule into a free radical trichloromethyl by the cytochrome P450 isozymes of the endoplasmic reticulum [32]. After the formation of trichloromethyl radical, it reacts with a molecular oxygen to form an even more highly toxic form known as the tri-chloromethoxy peroxyl radical [33]. The generated free radicals covalently bind to different proteins and lipids or by removing hydrogen atoms from the polyunsaturated fatty acids in lipids and thus initiate hepatic injury through the lipid peroxidation process. Thus, these unfolded chain of events result in the breakdown of the cellular membrane structure and disrupt the cellular energy process and protein synthesis [32]. As illustrated in Figure 5A, we observed that 5-DN significantly inhibited the production of ROS observed through H_2_DCFDA staining images and histogram. In addition, the production of lipid peroxidation marker malondialdehyde was remarkably downregulated in 5-DN treatment groups in comparison to the elevated levels observed in the CCl_4_ group (Figure 5E). Studies has shown MDA to be highly upregulated followed by a decrease in glutathione induced by the free radical metabolite carbon tetrachloride [34,35]. Likewise, as presented in Figure 5F,G, the CCl_4_ group exhibited reduced levels of antioxidant enzymes SOD and GSH, which significantly differed to those in the control group and 5-DN group in liver tissues. Furthermore, immunostaining and Western blot analysis indicated that 5-DN remarkably downregulated the expression of CYP2E1 (Figure 5B–D), which is a key metabolizing enzyme for CCl_4_ bio-activation, which was highly elevated in the CCl_4_ group, manifesting enhanced ROS generation and tissue damage.

### 2.5. 5-O-Demethylnobiletin Attenuates CCl_4_-Induced Liver Injury by Inhibiting Apoptosis

**Figure 6:**
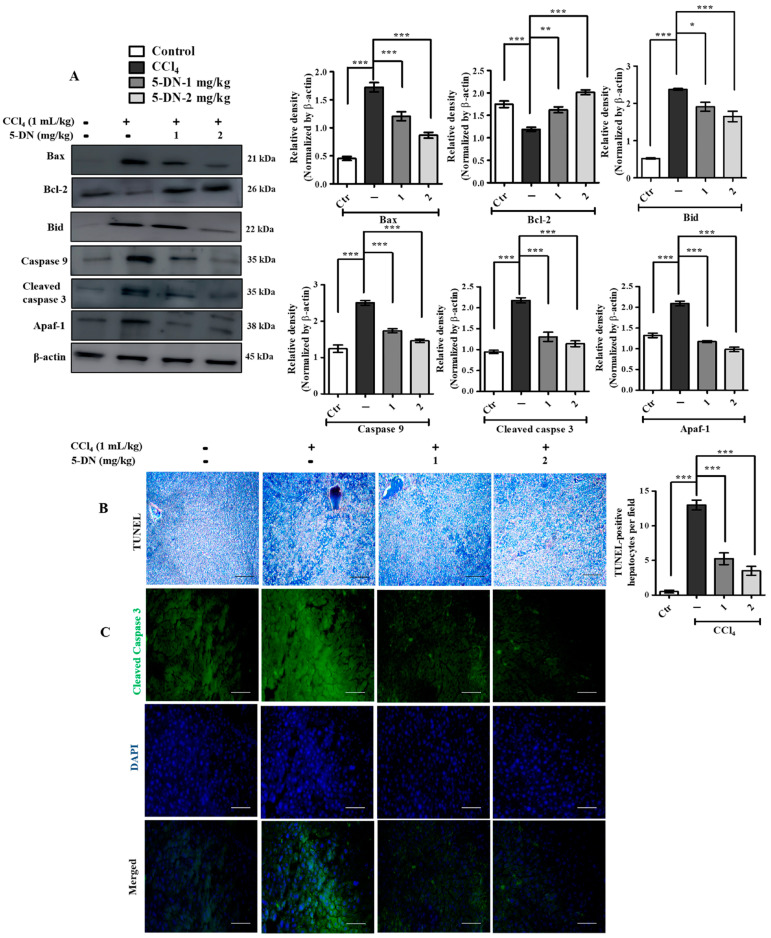
5-DN blockaded CCl_4_-induced apoptosis. (mice, n = 6/group). (**A**) Western blotting analysis of apoptosis markers and their quantification. (**B**) TUNEL assay to determine the occurrence of apoptosis. TUNEL-positive hepatocytes were counted from five different representative fields for all the groups and were evaluated and averaged for making the graph (Magnification: 200×). (**C**) Immunofluorescence staining of cleaved caspase 3 from BALB/c liver tissue (Magnification: 400×, Scheme 100 μM). Images were captured using Olympus BX51 fluorescence microscope. Densitometry analysis of these respective proteins were normalized by β-actin and evaluated through Image J software. The data are represented as mean ± S.D. of three independent experiments * *p* < 0.05, ** *p* < 0.01, *** *p* < 0.001 and ns (non-significant). Control vs. CCl_4_, CCl_4_ vs. 5-DN (1–2 mg/kg). Statistical significance analysis was carried out through one-way analysis of variance (ANOVA) prism.

CCl_4_ augments catastrophic liver damage by increasing the generation of ROS, genotoxicity, and DNA damage, thereby spurring necrotic and apoptotic events [36,37]. In order to enumerate whether CCl_4_ administration to mice would trigger apoptosis, we evaluated the expression of apoptosis-associated markers. CCl_4_ treatment significantly and markedly upregulated the protein expression of Bax, which is an important pro-apoptotic mitochondrial protein, and reduced the anti-apoptotic Bcl-2 expression. In addition, the expression of cleaved caspase 3, and caspase 9 was significantly reduced in the 5-DN treatment group in comparison to CCl_4_ treatment groups. Apaf-1, another important marker during the caspase-dependent mitochondrial apoptotic pathway [38], was noticeably and remarkably upregulated following CCl_4_ treatment, while 5-DN administration had lesser expression of an Apaf-1 protein (Figure 6A). We also performed immunofluorescence staining of cleaved caspase 3 on mice liver tissue (Figure 6C). The expression of cleaved caspase 3 was prominently higher in CCl_4_ treatment groups evaluated through the intensity of green fluorescence observed on the cytoplasmic regions of the liver tissue. In addition, Transferase-Mediated dUTP Biotin Nick End Labeling (TUNEL) assay staining (Figure 6B) revealed that CCl_4_ was able to drastically heighten the occurrence of apoptotic hepatocytes throughout the liver. The apoptotic hepatocytes were clearly and predominantly visible and located around the fibrous septa arising from the central veins and extending further. 5-DN treatment was able to significantly downregulate the expression of TUNEL-positive hepatocytes, indicating apoptotic blockade.

### 2.6. 5-O-Demethylnobiletin Promotes Autophagy in CCl_4_-Induced Liver Injurys

**Figure 7:**
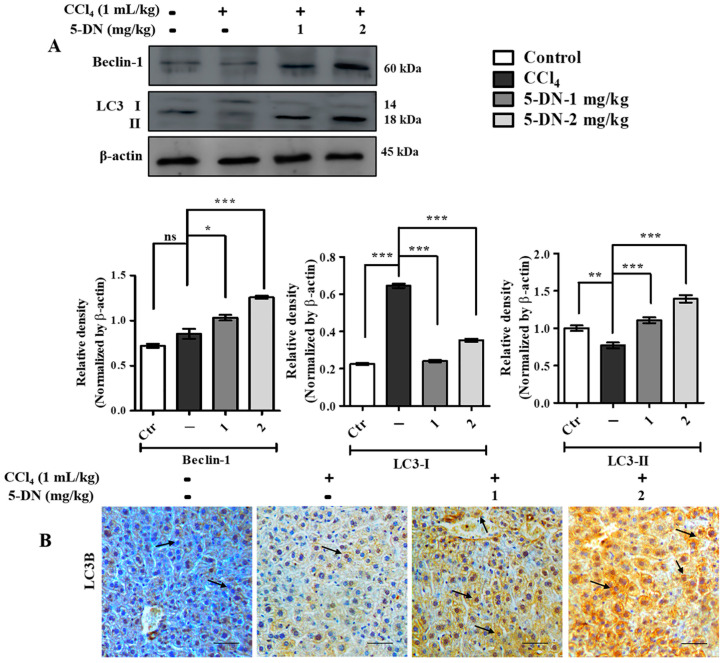
5-DN promoted autophagosome formation in CCl4-treated BALB/c mice (mice, n = 6/group). (A) Western blot-ting analysis of important autophagy markers and their quantification. (B) Immunohistochemical staining of LC3B from BALB/c liver tissue Arrow (→): indicates the formation of punctate structure in liver tissue. (Magnification: 200×, Scale bar (100 μM). Images were captured using an Olympus BX51 fluorescence microscope. Densitometry analysis of these respective proteins were normalized by β-actin and evaluated through Image J software. The data are represented as mean ± S.D. of three independent experiments * *p* < 0.05, ** *p* < 0.01, *** *p* < 0.001 and ns (non-significant). Control vs. CCl4, CCl4 vs. 5–DN (1–2 mg/kg). Statistical significance analysis was carried out through one-way analysis of variance (ANOVA) prism.

In order to confirm our experimental findings, we evaluated the different markers involved in autophagy. 5-DN treatment increased the expression of Beclin-1 in mice co-treated with CCl_4_ and 5-DN. In addition, 5-DN treatment had a higher conversion of LC3-I to LC3-II observed through Western blotting. Furthermore, we also performed immunohistochemistry for analyzing the concentration of LC3-II conversion by estimating LC3B. Since punctate LC3B staining is a major marker for substantiating the formation of autophagosomes and also as a confirmation for autophagy occurrence, we observed that 5-DN treatment groups had a prominently higher formation of punctate structure in comparison with the other groups (Figure 7B). These results revealed that 5-DN promoted autophagic flux in CCl_4_-treated mice.

## 3. Materials and Methods

### 3.1. Chemicals and Reagents

5-O-demethylnobiletin was purchased from Med-Chemexpress (Cat. No.: HY-N1942, purity > 99%). DMEM, fetal bovine serum (Gibco), penicillin–streptomycin, trypsin, DAPI (4′,6-diamidino-2-phenylindole), 3-(4,5-dimethylthiazol-2-yl)-2,5-diphenyltetrazolium bromide (MTT), dimethyl sulfoxide (DMSO), direct red, hematoxylin and eosin, and di-chlorodihydro-fluorescein diacetate (H_2_DCFDA) were purchased from Sigma-Aldrich, St. Louis, Missouri, USA. All other chemicals used for the experiment but not mentioned here were of the highest quality. The list of primary and secondary antibodies used for the study is also provided in the Supplementary Tables S1 and S2. The assay kits used for carrying out experiments for this study are mentioned in Supplementary Table S3.

### 3.2. Cell Viability Assay

For measuring cell survival, we referred to our previously published paper [39]. We evaluated the reduction of water-soluble yellow dye 3-(4,5 dimethylthiazol-2-yl)-2,5-diphenyltetrazolium bromide (MTT) (Sigma Aldrich: M2128) into an insoluble blue formazan product. Briefly, 96-well plates were used for seeding HepG2 cells at a density of 5 × 10^3^ cells/well and allowed to grow in a CO_2_ incubator. After the cells were 70% confluent, varying doses of CCl_4_ (5, 10, 20 mM) or 5-DN (10, 20, 40 μM) or in combination with CCl_4_ and 5-DN were used to treat the cells. Following 24 h incubation or different time points for CCl_4_-only treated groups, DMEM media was aspirated out and replaced with fresh 100 μL of DMEM in combination with 10 μL of MTT per well and was further incubated for 4 h at 37 °C. Later, the medium was aspirated out, and the insoluble formed formazan crystals were dissolved in 50 μL DMSO and incubated for 30 min in an ELISA plate shaker. The absorbance was measured at 540 nm using a microplate reader, and the percentage of cell proliferation was calculated as per the following formula: (absorbance of treated cells/absorbance of control cells) × 100. For performing LDH cytotoxicity assay, we followed the manufacturer’s protocol.

### 3.3. Cell Staining

HepG2 cells were cultured in DMEM media supplemented with 10% FBS and 1% penicillin–streptomycin at 37 °C under 5% CO_2_ incubation condition. For performing the experiment, cells were treated with CCl_4_ (20 mM) and 5-DN (20 and 40 μM) and incubated for 24 h. The cells were further used for fluorescence studies following the published protocols [40,41]. Acridine orange (AO) and ethidium bromide (EtBr) staining was followed for the detection of apoptotic cell formations and similarly JC-1 staining for mitochondrial membrane potential damage. After 24 h treatment with CCl4 and 5-DN, cells were washed with PBS and were fixed with cold 4% formaldehyde. Then, the fixed cells were washed again with PBS and treated with 1:1 ratio of AO/EtBr or JC-1 (20 μM) or H_2_DCFDA and incubated for 30 min at 37 °C. The cells were washed again with PBS, and the intensity of the resulting fluorescence was detected using an Olympus BX50 fluorescence microscope.

### 3.4. Acute Liver Damage Induction and Treatment

Male BALB/c mice (5–6 weeks) weighing around 25–30 g were purchased from Orient Bio (Seoul, South Korea) and housed under room temperature 22 ± 2 °C, 55 ± 5% humidity with 12 h light–dark cycle condition in the animal breeding center of Advanced Bio convergence center of Pohang Technopark foundation, South Korea. All the mice were provided with free access to drinking water and rodent food. Before the start of the study, we also ascertained that the experiments related to animal use were conducted in accordance with the Pohang Technopark Laboratory Ethics Committee (approval number ABCC2018004) after obtaining approval on 30 June 2018.

After one week of acclimatization, healthy mice were randomly allocated into six mice per group. For an acute liver injury model (n = 6/group), all mice were intraperitoneally injected with CCl_4_ (1 mL/kg) three times a week, except for control groups [42]. 5-DN mice groups were orally administered with 1 and 2 mg/kg every day until the end of the experiment. On the 15th day, mice were anesthetized with isoflurane, and the blood was drawn through cardiac puncture for biochemical analysis. Liver was weighed, measured, and collected for molecular and histological studies.

### 3.5. Serum Biochemical Examination

At the end of the experiment after 15 days, 1 mL of serum was collected per mouse through cardiac puncture. After blood collection, samples were incubated at room temperature for 30 min and then centrifuged at 14,000 rpm for 15 min. The supernatant was collected after centrifugation. Serum levels of aspartate aminotransferase (AST) and alanine aminotransferase (ALT) were measured using the previously described method [43] employing a chemistry analyzer (Mindary, WA, USA). Biochemical parameters were repeated three times.

### 3.6. Malondialdehyde Assay

For measuring lipid peroxidation, we estimated the concentration of malondialdehyde (MDA), which is the final product during a lipid peroxidation event estimated by following the manufacturer’s protocol (Abcam, Cambridge, CB2 0AX, UK). Optical density was measured at 532 nm through an ELISA plate reader (TECAN 200 infinite PRO).

### 3.7. Quantitative Determination of Inflammatory Markers through ELISA Assay

For the estimation of proinflammatory cytokines from liver, we homogenized the 30 mg of liver tissues and dissolved them with 600 μL of RIPA buffer and protease and phosphatase cocktail inhibitors, which were sonicated and incubated on ice for 20 min. The liver tissue homogenates were further centrifuged at 13,000× *g* for 10 min. The supernatant was collected, and levels of IL-6, TNF-α (R&D Systems Quantikine ELISA kits), GSH, and SOD (Biovision, Milpitas, CA, USA) were measured from the supernatants by the ELISA technique as per the manufacturer’s protocol (R&D Systems Quantikine ELISA kits). The details of the assay kits used for the experiment is provided in the Supplementary Table S3.

### 3.8. Intracellular ROS Production by H_2_DCHFDA Staining

For the detection of intracellular reactive oxygen species (ROS) production, we referred to a published paper [44], and the liver tissue paraffin embedded slides were incubated with different concentration of ice-cold methanol (5 min each) for proper permeabilization. After methanol treatment, PBST washing was given, and slides were immersed in acetone and incubated for a further 7 min. Later, distilled water washes (2–5 min) were given, and slides were blocked overnight at 4 °C in 3% BSA as a blocking agent. After that, we washed the slides with PDT 4 times (3 min each). After washing step, H_2_DCFDA (10 μM) was treated to each of the slides and incubated for 30 min at 37 °C. The ROS production was analyzed using an epi-fluorescence microscope Nikon Eclipse TS200 (Nikon Corp., Tokyo, Japan Japan) at 200× magnification. 

### 3.9. Hematoxylin and Eosin Staining

For performing H&E staining, we followed the protocol from our previously published paper [45]. The liver tissues were fixed with 4% paraformaldehyde solution for a few days. The liver tissues were processed for staining preparation by incubating them with different concentrations of ethanol and xylene and finally embedding in paraffin and sectioned at a thickness of 6 μM using a histocore rotary microtome. The slides were further allowed to dry for more than 6 h on a platform with a temperature adjusted to 5 °C. After drying the slides, they were immersed in xylene for deparaffinization. Next, the deparaffinized slides were dehydrated in different dilutions of EtOH (100, 90, 80, and 70%) for 3 min. The slides were washed on running tap water with care and immersed in hematoxylin for 15 min. Excessive stain was removed by washing it on running tap water. The slides were again immersed in a 1% HCl-EtOH solution for 30 s, washed with water and immersed on ammonia water for another 30 sec, and then washed again with water. Slides were immersed with eosin for 2 min to counter-stain, which was followed by a dehydration process of ethanol and xylene fixation in a reverse order performed at the beginning of the staining process. A coverslip was added onto the slides along with a few drops of mounting medium and analyzed under a light microscope (200× resolution).

### 3.10. Prussian Blue Stain

We followed the previously published paper for performing Prussian blue staining [46]. Liver tissue were stored in 10% paraformaldehyde, embedded in paraffin, and sectioned at 5 μM. The slides were de-paraffinized and hydrated in distilled water. Equal parts of mixed hydrochloric acid and potassium ferrocyanide were mixed and prepared. Slides were immersed in the prepared solution and incubated for 20 min. The slides were washed with distilled water, and the slides were dehydrated using different concentrations of alcohol (95% and 100%) for 3 min each. Later, the slides were dipped in xylene (2×) for a duration of 3 min. The slides were covered using a coverslip and a mounting medium. The slides were viewed under a microscope for the accumulation of iron deposits.

### 3.11. Picrosirius Red Staining for Detection of Collagen Histochemistry

For performing the detection of collagen histochemistry, we followed the Picrosirius red staining method. The paraffin-embedded slides were de-waxed in xylene solution and dehydrated in EtOH solution. Next, the slides were stained with hematoxylin (10 min) and washed under running tap water for another 10 min. The slides were incubated with direct red solution for 1 h. After Sirius red staining, the slides were washed with two changes of acidified water, which was followed by vigorous shaking to remove any remaining acidified water on them. Later, the slides were dehydrated by incubating in 100% ethanol for 5 min. Finally, the slides were cleared in xylene and covered with a coverslip after adding a few drops of mounting medium. The slides were viewed under a microscope for accumulation of collagen deposits.

### 3.12. Immunohistochemistry

For performing immunohistochemistry, the liver tissues were processed for tissue preparation as discussed earlier. Before the start of the staining process, the slides were heated by boiling for the antigen retrieval step. The sectioned slides (8 μM) were immersed in xylene (5 min, 2×). After xylene fixation, slides were dehydrated in 100%, 95%, 80%, 70%, and 50% EtOH (5 min each). After the end of the dehydration step, slides were washed with PBS (3 min, 3×) and were further incubated with a blocking buffer solution (SuperBlock, Thermofisher Scientific). After blocking, slides were treated with primary antibodies such as αSMA, CYP2E1, and LC3B [47] (Bioworld Technology) and incubated overnight at 4 °C. After primary antibody incubation, slides were washed with PBS and incubated with secondary antibody for 30 min at room temperature. After secondary antibody treatment on the slides, a few drops of DAB substrate solution were added onto each slides and were further incubated for 15 min. Following DAB incubation, slides were washed with distilled water and counter-stained by the nucleus using hematoxylin for 1 min. The slides were incubated with different concentrations of EtOH followed by xylene incubation following the protocol performed during the start of the experiment. Finally, slides were fixed using a mounting medium and allowed to air dry properly, removing unwanted bubbles from them. Then, the slides were observed under a microscope for data collection (Olympus BX51 Fluorescence Microscope).

### 3.13. Immunofluorescence Staining

For performing immunofluorescence staining, we referred to our previously published paper [48], paraffin-embedded liver tissues were dewaxed by incubating them in xylene and dehydrated using different concentration of EtOH. Next, we performed a heat- induced antigen retrieval step using a pH 9.5 buffer (0.1 M TRIS/HCl and 5% urea) and heating the coverslip at 95 °C for 10 min. The slides were pre-rinsed with PBS (5 min, 3×). Next, samples were incubated with PBS containing 0.1% triton X-100 (10 min) to help in the penetration of the antibody. Later, the slides were washed with PBS for 5 min. Next, the slides were incubated with 1% BSA containing glycine in PBST (PBS + 0.1% tween 20) for 30 min so as to block any unspecific antibody binding and to avoid high background staining. After blocking, the slides were incubated with cleaved caspase 3 [49] and dissolved in 1% BSA in PBST in a humidified chamber overnight at 4 °C. The next day, the slides were washed in PBS (5 min, 3×). After the washing step, the slides were further incubated with FITC-secondary antibody and dissolved in 1% BSA for 1–3 h at room temperature in a dark compartment to avoid loss of fluorescence. After the desired incubation, the FITC-secondary antibody solution was decanted, and the slides were washed again with PBS (5 min, 3×) in the dark. Later, the slides were counterstained with DAPI or Hoechst (DNA stain) at a concentration of 1 μg/mL for 15 min in the dark. The slides were rinsed with PBS and mounted with a coverslip by adding a few drops of mounting medium. The coverslips were later sealed using a nail polish to further prevent the slide from drying. Images were observed under a microscope (Olympus BX51 Fluorescence Microscope) at 200×.

### 3.14. Measurement of Apoptotic Hepatocytes Using Terminal Deoxynucleotidyl Transferase-Mediated dUTP Biotin Nick End Labeling (TUNEL) Assay

For the detection of apoptosis in paraffin-embedded liver tissues, we performed the experiment as per the manufacturer’s protocol using a TUNEL peroxidase detection kit (DeadEnd™ Colorimetric TUNEL System, Promega). The slides were washed in xylene (5 min, twice) and immersed in decreasing concentration of ethanol (100%, 95%, 85%, 70%, and 50%) for 3 min each. Next, slides were immersed in 0.85% NaCl (5 min) and washed with PBS (5 min). Then, the slides were immersed in 4% paraformaldehyde (15 min) and washed with PBS (5 min, twice). Next, for the permeabilization step, we added 100 μL of 20 μg/mL proteinase K solution followed by incubation at room temperature for 30 min. Later, slides were washed in PBS (5 min) and fixed again with 4% paraformaldehyde (5 min). Slides were washed in PBS (5 min, twice) and incubated in 100 μL equilibration buffer (10 min). Next, 100 μL of TdT reaction mix was added to the tissue sections on the slides and cover slides with plastic coverslips followed by incubation for 1 h at 37 °C. Later, the plastic coverslips were removed, and the slides were immersed in 2 × SSC for 15 min. Slides were re-washed in PBS (5 min, twice) and immersed in 0.3% hydrogen peroxide (5 min). The slides were washed in PBS (5 min), 100 μL of streptavidin HRP (1:500 in PBS) was added to each slide, and the slides were incubated for 30 min at room temperature. Then, the slides were again washed in PBS (5 min) and incubated in DAB solution until a light brown background appeared. Finally, the slides were washed several times in distilled water and mounted with a medium with coverslips and observed under a microscope.

### 3.15. Western Blotting 

The liver tissue was homogenized following the previously mentioned protocol in Section 3.7. The concentration of the protein was measured through Bradford assay at 595 nm wavelength. Proteins were loaded onto an SDS-polyacrylamide gel along with a reference protein ladder [50]. After the completion of protein separation, the gel was transferred onto a PVDF membrane. The membranes were further incubated in 3% BSA prepared in TBST (1 h) for blocking of the membranes. After incubation, the membranes were washed with TBST (5 min, 3×) and were treated with primary antibody against the protein of interest. To visualize, the membranes were incubated for 3 h at 37 °C or overnight at 4 °C in a gel rocker. After that, membranes were washed with TBST (5 min, 3×) followed by treatment with secondary antibody (2 h) at room temperature in a gel rocker. The membranes were again washed with TBST (3 times, 5 min each). The membranes were visualized by using enhanced chemiluminescence Western blotting detection reagents (Amersham Biosciences Inc., Piscataway, NJ, USA).

### 3.16. Statistical Analysis

For determining the quantitative results of all the experiments performed in this study, the values are expressed as a mean ± standard deviation (SD) of the experiments. The statistical significance and the differences in the experimental groups were calculated by using one-way analysis of variance (ANOVA) with Tukey’s test comparing all pair of columns and Dunnett’s post-comparison test for multiple groups, where * represents *p*-values < 0.05, ** represents *p*-values < 0.01, and *** represents *p*-values < 0.001.

## 4. Conclusions

**Figure 8:**
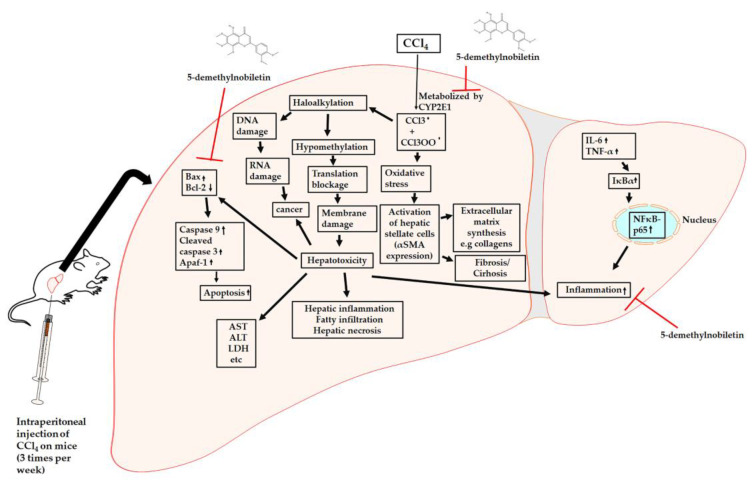
Role of 5-DN in counteracting CCl4-induced acute liver injury. (↑): represents upregulation of the spe-cific marker in response to CCl4 or 5-DN. (↓): represents downregulation of the specific marker in response to CCl4 or 5-DN. (┴): represents inhibition after treatment with 5-DN.

Our findings from this study clearly showed that 5-DN was able to attenuate CCl_4_-induced fibrotic liver and effectively suppressed inflammatory rush on the liver tissue, by inhibiting of IκBα and NF-κB-p65 and the subsequent drop in the pro-inflammatory cytokine production (Figure 8). 5-DN also effectively downregulated the expression of CYP2E1 and inhibited apoptosis arising from CCl_4_-induced hepatic damage and subsequently promoting the autophagic flux. Conclusively, our findings from this study point toward the very strong positive effects 5-DN had on acute liver injury, although further studies need to be carried out to validate the effectiveness of 5-DN on fibrosis. 

## Supplementary Materials

The following are available online at https://www.mdpi.com/1422-0067/22/3/1083/s1.

## References

[1] Rowland A., Miners J.O., Mackenzie P.I. (2013). The UDP-glucuronosyltransferases: Their role in drug metabolism and detoxification. Int. J. Biochem. Cell Biol..

[2] Friedman S.L. (2003). Liver fibrosis—From bench to bedside. J. Hepatol. Suppl..

[3] Jansen P.L.M. (2004). Non-alcoholic steatohepatitis. Eur. J. Gastroenterol. Hepatol..

[4] Maher J.J., McGuire R.F. (1990). Extracellular matrix gene expression increases preferentially in rat lipocytes and sinusoidal endothelial cells during hepatic fibrosis In Vivo. J. Clin. Investig..

[5] Friedman S.L. (2000). Molecular regulation of hepatic fibrosis, an integrated cellular response to tissue injury. J. Biol. Chem..

[6] Karsdal M.A., Nielsen S.H., Leeming D.J., Langholm L.L., Nielsen M.J., Manon-Jensen T., Siebuhr A., Gudmann N.S., Rønnow S., Sand J.M. (2017). The good and the bad collagens of fibrosis—Their role in signaling and organ function. Adv. Drug Deliv. Rev..

[7] Friedman S.L., Rockey D.C., McGuire R.F., Maher J.J., Boyles J.K., Yamasaki G. (1992). Isolated hepatic lipocytes and kupffer cells from normal human liver: Morphological and functional characteristics in primary culture. Hepatology.

[8] Eng F.J., Friedman S.L. (2000). Fibrogenesis I. New insights into hepatic stellate cell activation: The simple becomes complex. Am. J. Physiol. Gastrointest. Liver Physiol..

[9] Dong R., Luo Y., Zheng S. (2012). α-SMA overexpression associated with increased liver fibrosis in infants with biliary atresia. J. Pediatr. Gastroenterol. Nutr..

[10] Malhi H., Kaufman R.J. (2011). Endoplasmic reticulum stress in liver disease. J. Hepatol..

[11] Klaassen C.D., Plaa G.L. (1967). Relative effects of various chlorinated hydrocarbons on liver and kidney function in dogs. Toxicol. Appl. Pharmacol..

[12] Huang H.L., Wang Y.J., Zhang Q.Y., Liu B., Wang F.Y., Li J.J., Zhu R.Z. (2012). Hepatoprotective effects of baicalein against CCl4-induced acute liver injury in mice. World J. Gastroenterol..

[13] Chen X., Meng Q., Wang C., Liu Q., Sun H., Huo X., Sun P., Yang X., Peng J., Liu K. (2015). Protective effects of calycosin against CCl4-induced liver injury with activation of FXR and STAT3 in mice. Pharm. Res..

[14] Basu S. (2011). Carbon Tetrachloride-Induced Hepatotoxicity: A Classic Model of Lipid Peroxidation and Oxidative Stress. Studies on Experimental Models.

[15] Weber L.W.D., Boll M., Stampfl A. (2003). Hepatotoxicity and mechanism of action of haloalkanes: Carbon tetrachloride as a toxicological model. Crit. Rev. Toxicol..

[16] Caetano-Anollés G., Yafremava L.S., Gee H., Caetano-Anollés D., Kim H.S., Mittenthal J.E. (2009). The origin and evolution of modern metabolism. Int. J. Biochem. Cell Biol..

[17] Kumar S., Pandey A.K. (2013). Chemistry and biological activities of flavonoids: An overview. Sci. World J..

[18] Tirkey N., Pilkhwal S., Kuhad A., Chopra K. (2005). Hesperidin, a citrus bioflavonoid, decreases the oxidative stress produced by carbon tetrachloride in rat liver and kidney. BMC Pharmacol..

[19] Dong D., Xu L., Yin L., Qi Y., Peng J. (2015). Naringin prevents carbon tetrachloride-induced acute liver injury in mice. J. Funct. Foods.

[20] Omar H.A., Mohamed W.R., Arab H.H., Arafa E.S.A. (2016). Tangeretin alleviates cisplatin-induced acute hepatic injury in rats: Targeting MAPKs and apoptosis. PLoS ONE.

[21] Guo S., Zhang Y., Wu Z., Zhang L., He D., Li X., Wang Z. (2019). Synergistic combination therapy of lung cancer: Cetuximab functionalized nanostructured lipid carriers for the co-delivery of paclitaxel and 5-demethylnobiletin. Biomed. Pharmacother..

[22] Guo R., Lin B., Pan J.F., Liong E.C., Xu A.M., Youdim M., Fung M.L., So K.F., Tipoe G.L. (2016). Inhibition of caspase-9 aggravates acute liver injury through suppression of cytoprotective autophagy. Sci. Rep..

[23] Zhao X., Fu J., Xu A., Yu L., Zhu J., Dai R., Su B., Luo T., Li N., Qin W. (2015). Gankyrin drives malignant transformation of chronic liver damage-mediated fibrosis via the Rac1/JNK pathway. Cell Death Dis..

[24] Kyriakis J.M., Avruch J. (2001). Mammalian mitogen-activated protein kinase signal transduction pathways activated by stress and inflammation. Physiol. Rev..

[25] Schulze-Osthoff K., Ferrari D., Riehemann K., Wesselborg S. (1997). Regulation of NF-κB activation by MAP kinase cascades. Immunobiology.

[26] Kaminska B. (2005). MAPK signalling pathways as molecular targets for anti-inflammatory therapy—From molecular mechanisms to therapeutic benefits. Biochim. Biophys. Acta Proteins Proteom..

[27] Ji L., Xue R., Tang W., Wu W., Hu T., Liu X., Peng X., Gu J., Chen S., Zhang S. (2014). Toll like receptor 2 knock-out attenuates carbon tetrachloride (CCl_4_)-induced liver fibrosis by downregulating MAPK and NF-κB signaling pathways. FEBS Lett..

[28] Marra F., Arrighi M.C., Fazi M., Caligiuri A., Pinzani M., Romanelli R.G., Efsen E., Laffi G., Gentilini P. (1999). Extracellular signal-regulated kinase activation differentially regulates platelet-derived growth factor’s actions in hepatic stellate cells, and is induced by In Vivo liver injury in the rat. Hepatology.

[29] Kluwe J., Pradere J.P., Gwak G.Y., Mencin A., De Minicis S., Österreicher C.H., Colmenero J., Bataller R., Schwabe R.F. (2010). Modulation of Hepatic Fibrosis by c-Jun-N-Terminal Kinase Inhibition. Gastroenterology.

[30] Wang M., Meng D., Zhang P., Wang X., Du G., Brennan C., Li S., Ho C.T., Zhao H. (2018). Antioxidant protection of nobiletin, 5-demethylnobiletin, tangeretin, and 5-demethyltangeretin from citrus Peel in Saccharomyces cerevisiae. J. Agric. Food Chem..

[31] Liang F., Fang Y., Cao W., Zhang Z., Pan S., Xu X. (2019). Tangeretin attenuates tert-Butyl Hydroperoxide (tBHP)-induced oxidative damage in HepG2 cells: Relevance of Nrf2/ARE and MAPKs signaling pathways. J. Agric. Food Chem..

[32] Recknagel R.O., Glende E.A., Dolak J.A., Waller R.L. (1989). Mechanisms of carbon tetrachloride toxicity. Pharmacol. Ther..

[33] Williams A.T., Burk R.F. (1990). Carbon tetrachloride hepatotoxicity: An example of free radical-mediated injury. Semin. Liver Dis..

[34] Galicia-Moreno M., Rosique-Oramas D., Medina-Avila Z., Álvarez-Torres T., Falcón D., Higuera-De La Tijera F., Béjar Y.L., Cordero-Pérez P., Muñoz-Espinosa L., Pérez-Hernández J.L. (2016). Behavior of oxidative stress markers in alcoholic liver cirrhosis patients. Oxid. Med. Cell. Longev..

[35] Connor H.D., Lacagnin L.B., Knecht K.T., Thurman R.G., Mason R.P. (1990). Reaction of glutathione with a free radical metabolite of carbon tetrachloride. Mol. Pharmacol..

[36] Lin S.Y., Dan X., Du X.X., Ran C.L., Lu X., Ren S.J., Tang Z.T., Yin L.Z., He C.L., Yuan Z.X. (2019). Protective effects of salidroside against carbon tetrachloride (Ccl4)-induced liver injury by initiating mitochondria to resist oxidative stress in mice. Int. J. Mol. Sci..

[37] Alkreathy M.M., Khan A.A., Khan R.R., Sahreen S. (2014). CCl_4_ induced genotoxicity and DNA oxidative damages in rats: Hepatoprotective effect of Sonchus arvensis. BMC Complement. Altern. Med..

[38] Brentnall M., Rodriguez-Menocal L., De Guevara R.L., Cepero E., Boise L.H. (2013). Caspase-9, caspase-3 and caspase-7 have distinct roles during intrinsic apoptosis. BMC Cell Biol..

[39] Dey D.K., Koo B.G., Sharma C., Kang S.C. (2019). Characterization of Weissella confusa DD_A7 isolated from kimchi. LWT-Food Sci. Technol..

[40] Dey D.K., Chang S.N., Vadlamudi Y., Park J.G., Kang S.C. (2020). Synergistic therapy with tangeretin and 5-fluorouracil accelerates the ROS/JNK mediated apoptotic pathway in human colorectal cancer cell. Food Chem. Toxicol..

[41] Dey D.K., Kang S.C. (2020). Aflatoxin B1 induces reactive oxygen species-dependent caspase-mediated apoptosis in normal human cells, inhibits *Allium cepa* root cell division, and triggers inflammatory response in zebrafish larvae. Sci. Total Environ..

[42] Bencheikh N., Bouhrim M., Kharchoufa L., Choukri M., Bnouham M., Elachouri M. (2019). Protective effect of zizyphus lotus L. (Desf.) fruit against CCl_4_-induced acute liver injury in rat. Evid. Based Complement. Altern. Med..

[43] Lee E.Y., Kim S.H., Chang S.N., Lee J.H., Hwang B.S., Woo J.T., Kang S.C., Lee J., Park J.G. (2019). Efficacy of polymethoxylated flavonoids from *Citrus depressa* extract on alcohol-induced liver injury in mice. Biotechnol. Bioprocess. Eng..

[44] Sharma C., Kang S.C. (2020). Garcinol pacifies acrylamide induced cognitive impairments, neuroinflammation and neuronal apoptosis by modulating GSK signaling and activation of pCREB by regulating cathepsin B in the brain of zebrafish larvae. Food Chem. Toxicol..

[45] Chang S.N., Khan I., Dey D.K., Cho K.H., Hwang B.S., Bae K.B., Kang S.C., Park J.G. (2019). Decursinol angelate ameliorates 12-O-tetradecanoyl phorbol-13-acetate (TPA)-induced NF-κB activation on mice ears by inhibiting exaggerated inflammatory cell infiltration, oxidative stress and pro-inflammatory cytokine production. Food Chem. Toxicol..

[46] Li X., Wei Z., Lv H., Wu L., Cui Y., Yao H., Li J., Zhang H., Yang B., Jiang J. (2019). Iron oxide nanoparticles promote the migration of mesenchymal stem cells to injury sites. Int. J. Nanomed..

[47] Lazova R., Camp R.L., Klump V., Siddiqui S.F., Amaravadi R.K., Pawelek J.M. (2012). Punctate LC3B expression is a common feature of solid tumors and associated with proliferation, metastasis, and poor outcome. Clin. Cancer Res..

[48] Dey D.K., Chang S.N., Kang S.C. (2021). The inflammation response and risk associated with aflatoxin B1 contamination was minimized by insect peptide CopA3 treatment and act towards the beneficial health outcomes. Environ. Pollut..

[49] Li Y., Xia Y., Cheng X., Kleiner D.E., Hewitt S.M., Sproch J., Li T., Zhuang H., Jake Liang T. (2019). Hepatitis B surface antigen activates unfolded protein response in forming ground glass hepatocytes of chronic hepatitis B. Viruses.

[50] Chang S.N., Dey D.K., Oh S.T., Kong W.H., Cho K.H., Al-Olayan E.M., Hwang B.S., Kang S.C., Park J.G. (2020). Phorbol 12-myristate 13-acetate induced toxicity study and the role of tangeretin in abrogating HIF-1α-NF-κB crosstalk In Vitro and In Vivo. Int. J. Mol. Sci..

