# Supplementary material for: 5-O-Demethylnobiletin Alleviates CCl4-Induced Acute Liver Injury by Equilibrating ROS-Mediated Apoptosis and Autophagy Induction
Source: Int J Mol Sci. 2021 Jan 22;22(3):1083. doi: 10.3390/ijms22031083 (PMC7865239; doi:10.3390/ijms22031083)
Supplement: Supplementary file 1 [file ijms-22-01083-s001.pdf]

Supplementary Information

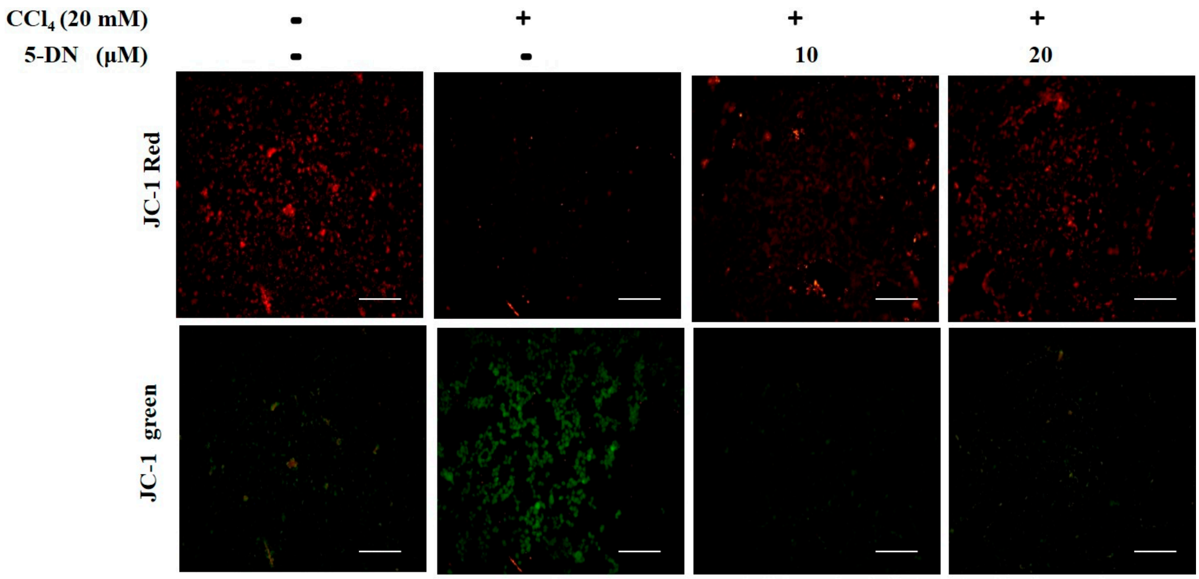

Figures S1. JC-1 staining on HepG2 cells to determine the extent of mitochondrial membrane damage.

Table S1. List of primary antibodies used in the study.

S.No.	Protein name	Company name	Molecular weight	Dilution	Host	Secondary antibody
1	iNOS (ab3523)	Abcam	135	1:200	Rabbit	Goat anti rabbit-HRP
2	p-ERK 1/2 (sc-16982)	Santa cruz	42/44	1:50-1:500	Rabbit	Donkey anti goat-HRP
3	p-p38 (sc-17582-R)	Santa cruz	38	1:50-1:500	Rabbit	Goat anti rabbit-HRP
4	IκBα (BS90963)	Bioworld	34	1:1000 1:5000	Rabbit	Goat anti rabbit-HRP
5	NF-κB p65 (#8242)	Cell signaling	65	1:100-1:1000	Rabbit	Goat anti rabbit-HRP
6	Anti-cytochrome p450 2E1 (ab28146)	Abcam	50-55	1:5000	Rabbit	Goat anti rabbit-HRP
7	Bcl-2 (ALX-201-701)	ENZO	26	1:1000	Rabbit	Goat anti rabbit-HRP
8	Bax (#2772S)	Cell signaling	21	1:1000	Rabbit	Goat anti rabbit-HRP
9	Caspase 9 (Adl-Aap-109)	ENZO	35,47,48	1:1000	Rabbit	Goat anti rabbit-HRP
10	Caspase 3 (BS6428)	Bioworld	35	1:1000 1:5000	Rabbit	Goat anti rabbit-HRP
11	Cleaved caspase 3	Cell signaling	17,19	1:1000	Rabbit	Goat anti rabbit-HRP
12	Bid (sc-11423)	Santa cruz	22	1:50-1:500	Rabbit	Donkey anti goat-HRP
13	Apaf-1 (BS1016)	Bioworld	38	1:1000 1:5000	Rabbit	Goat anti rabbit-HRP
14	Beclin-1 (3495S)	Cell signaling	60	1:1000	Rabbit	Goat anti rabbit-HRP
15	Beclin-1 (3495S)	Cell signaling	60	1:1000	Rabbit	Goat anti rabbit-HRP
16	ATG-7 (8558S)	Cell signaling	78	1:1000	Rabbit	Goat anti rabbit-HRP
17	LC3 A/B (#12741)	Cell signaling	14,18	1:1000	Rabbit	Goat anti rabbit-HRP
18	β-actin (#4967)	Cell signaling	45	1:1000	Rabbit	Goat anti rabbit-HRP

Table S2. List of secondary antibodies used in the study.

S.No.	Name of secondary antibody	Company name	Dilution	Reactivity
1	Goat anti rabbit-HRP (NBP2-30348H)	Novus Biologicals (Bethyl)	1:5000	Anti-rabbit
2	Donkey anti goat-HRP (NBP2-68552)	Novus Biologicals (Bethyl)	1:5000	Anti-goat
3	Goat anti mouse-HRP	Santa cruz	1:2000-1:10000	Anti-mouse
4	Goat anti rabbit-FITC	Santa cruz	1:100 1:400	Anti-rabbit

Table S3. List of ELISA assay kits used in the study.

S.No.	Name of secondary antibody	Company name
1	Mouse IL-6 (#M6000B)	Quantikine ELISA
2	Mouse TNF-α (#MTA00B)	Quantikine ELISA
3	Lipid Peroxidation (MDA) Assay kit (ab118970)	Abcam
4	LDH cytotoxicity Assay Kit (CAT# KTA1030)	Abbkine
5	Glutathione assay Kit (K261-100)	BioVision
6	Superoxide Dismutase (SOD) Activity Colorimetric Assay Kit (K335-100)	BioVision
7	DeadEnd™ Colorimetric TUNEL System	Promega
